# Special Issue on “Machine Learning-Aided Medical Image Analysis”

**DOI:** 10.3390/bioengineering13030273

**Published:** 2026-02-27

**Authors:** Amirreza Mahbod, Ramona Woitek

**Affiliations:** Research Center for Medical Image Analysis and Artificial Intelligence, Department of Medicine, Faculty of Medicine and Dentistry, Danube Private University, 3500 Krems an der Donau, Austria; ramona.woitek@dp-uni.ac.at

## 1. Introduction

With the advancement of computers and digitalization, many medical records and data, including medical images, can now be stored and analyzed using semi- or fully automated computerized approaches. Considering the increasing volume of medical data and the shortage of medical experts in different parts of the world, the need for accurate and reliable automated methods to support clinical decision-making has become more evident. This is particularly important for medical images across different modalities, ranging from histopathological to radiological imaging.

While many approaches have been proposed for medical image analysis, machine learning (ML) and deep learning (DL) methods have demonstrated superior performance compared to traditional techniques, especially in complex and large-scale datasets. ML- and DL-based approaches can be applied to various tasks such as feature extraction, classification, detection, segmentation, registration, and image retrieval, enabling numerous downstream clinical applications [1,2].

The aim of this Special Issue was to highlight recent advances in emerging ML and DL methodologies, with a particular focus on medical image analysis. This Special Issue includes six selected papers covering a wide range of ML applications across diverse medical imaging tasks and domains. All articles underwent a rigorous peer review process involving at least two independent reviewers and two rounds of revisions.

## 2. Papers Included in the Special Issue

This section provides a brief description of the articles published in this Special Issue. The included studies cover various aspects and applications of ML and DL in medical image analysis, including sample quality control (QC) [3], image classification [4,5], super-resolution [6], anatomical landmark localization [7], and other related tasks across different types of medical images, such as histological images [3,4], RGB-D images with clinical applications [8], and radiological images [5,6,7].

The developed and applied methodological approaches were based on various techniques, including the use of foundation models [3,4], fine-tuned convolutional neural networks (CNNs) [4,5,6,7,8] or vision transformers (ViTs) [4], as well as classical machine learning models [3]. A brief description of each article (ordered according to the publication dates) is presented below.

In the study titled “PathQC: Determining Molecular and Structural Integrity of Tissues from Histopathological Slides,” Sinha et al. [3] proposed a regression-based framework to predict RNA Integrity Number (RIN) and autolysis scores—established indicators of molecular and structural tissue quality—directly from Hematoxylin and Eosin (H&E)-stained whole-slide images. Their approach involved preprocessing whole-slide images through patch extraction, followed by feature extraction using a digital pathology foundation model. Patch-level features were then aggregated to generate a slide-level embedding representing the entire whole-slide image. Finally, a supervised Lasso regression model was trained to predict RIN and autolysis scores from these embeddings. Although the model demonstrated variable performance across different tissue types, it showed strong predictive performance in certain tissues, such as adrenal gland and colon. Despite variability across tissues, the approach remained valuable as a non-destructive screening tool to filter out low-quality samples prior to downstream molecular analyses.

In the study titled “Integrating Foundation Model Features into Graph Neural Network and Fusing Predictions with Standard Fine-Tuned Models for Histology Image Classification”, Saeidi et al. [4] proposed a lightweight graph neural network (GNN) that incorporated pathology foundation model features as node representations for the task of histological image patch classification. Their results demonstrated that leveraging foundation model features outperformed conventional pre-trained CNNs or ViTs features and delivered performance comparable to end-to-end fine-tuning of CNNs. The best overall classification performance was achieved using a fusion strategy, where predictions from the best-performing GNN and fine-tuned CNN model were combined using a weighted averaging approach.

In the study titled “Automated Mucormycosis Diagnosis from Paranasal CT Using ResNet50 and ConvNeXt Small”, Toprak et al. [5] proposed a standard fine-tuning approach based on ResNet50 and ConvNeXt-Small models for a novel clinical application, namely the diagnosis of mucormycosis from computed tomography (CT) images by classifying them into three categories: mucormycosis, polyp, and normal. Their results demonstrated strong classification performance for both models based on well-established evaluation metrics. To enhance interpretability, the authors employed the Grad-CAM approach to highlight the regions of the images that contributed most to the model’s predictions, thereby providing more interpretable and clinically meaningful results.

In the study titled “The Feasibility of RGB-D Gaze Intention Measurement in Children with Autism Using Azure Kinect”, Bendimered et al. [8] proposed a gaze tracking method for estimating gaze direction in children with autism spectrum disorder (ASD). Their method was based on two main models. The first model tracked eye movements at short distances, while the second model estimated head orientation at longer distances. For the first model, they used data and features provided by the Azure Kinect camera together with the GazeML machine learning approach. For the second model, they relied on the 3D joint data provided by the Azure Kinect camera and applied geometric calculations to estimate head orientation. Their experimental results, conducted both on adults for initial validation and on two children with autism, demonstrated robust and accurate performance of the proposed model for gaze tracking. This has clinical relevance, as reduced eye contact, which can be measured through gaze estimation, is an early marker of ASD.

In the study titled “FDoSR-Net: Frequency-Domain Informed Auto-Encoder Network for Arbitrary-Scale 3D Whole-Heart MRI Super-Resolution”, Maciel et al. [6] proposed a DL–based super-resolution framework for converting low-resolution 3D whole-heart MRI volumes into high-resolution images with arbitrary scaling factors between 2× and 4×. Unlike conventional encoder–decoder autoencoder architectures, their model incorporated a frequency-domain–informed regularization term into the loss function. This regularizer operated in the synthetic Fourier domain by masking selected regions of the frequency spectrum and penalizing discrepancies between the ground-truth and reconstructed images to enhance the preservation of fine structural details during super-resolution. The proposed method was evaluated using multiple reconstruction metrics and demonstrated competitive or superior performance relative to conventional nearest-neighbor interpolation and other state-of-the-art super-resolution methods.

In the study titled “Implicit Is Not Enough: Explicitly Enforcing Anatomical Priors inside Landmark Localization Models”, Joham et al. [7] proposed a framework for robust anatomical landmark localization in hand X-ray images. While conventional U-Net-based models primarily rely on implicit anatomical constraints embedded in the network architecture or loss formulation, the authors introduced the Global Anatomical Feasibility Filter and Analysis (GAFFA), a differentiable refinement module that explicitly incorporated anatomical priors through a Markov Random Field (MRF) formulation. In this framework, each landmark was represented as a node in a graph reflecting natural bone connectivity, and pairwise anatomical relationships were modeled via conditional spatial distributions learned from training data. GAFFA performed a differentiable approximation of a single iteration of the sum-product algorithm to refine the initial U-Net heatmap predictions. The final landmark coordinates were then extracted using a Differentiable Spatial to Numerical Transform (DSNT) and optimized with coordinate-level mean squared error supervision. Experimental evaluation on a public hand X-ray dataset demonstrated improved robustness to large outliers and challenging occlusion scenarios compared with approaches relying solely on implicit refinement strategies.

## 3. Conclusions

As demonstrated by the studies presented in this editorial, the application of ML in medical image analysis is broad and continuously expanding. There is a clear trend toward utilizing more advanced architectures to automate various tasks in medical image analysis using state-of-the-art ML and DL approaches. In this editorial, we briefly highlighted several applications that employed diverse methodological approaches in the field of medical image analysis. This field remains open and highly promising for novel and innovative research in the future.

As a final note, we would like to thank all authors for submitting their papers to this Special Issue and for revising their manuscripts to enhance their quality based on reviewers’ suggestions. We also sincerely thank all reviewers for their valuable support and constructive feedback. Finally, we would like to express our appreciation to the *Bioengineering* support team for handling all administrative and publication requirements.

## Data Availability

Not applicable.

## Abbreviations

The following abbreviations are used in this manuscript:

ML	Machine Learning
DL	Deep Learning
QC	Quality Control
CNN	Convolutional Neural Network
ViT	Vision Transformer
RIN	RNA Integrity Number
H&E	Hematoxylin and Eosin
GNN	Graph Neural Network
MRI	Magnetic Resonance Imaging
CT	Computed Tomography
ASD	Autism Spectrum Disorder
GAFFA	Global Anatomical Feasibility Filter and Analysis
MRF	Markov Random Field
DSNT	Differentiable Spatial to Numerical Transform
